# Determination of Acylglycerols in Diesel Oils by GC

**DOI:** 10.1080/15376510701624068

**Published:** 2008-06-23

**Authors:** Rafał Wawrzyniak, Wiesław Wasiak

**Affiliations:** Faculty of Chemistry, Adam Mickiewicz University Poznań, Poland

**Keywords:** Acylglycerols, Cooking Oil, Diesel Oil, FAME (Fatty Acid Methyl Ester), Free Fatty Acids, GC

## Abstract

In many EU countries and outside the EU, besides the addition of pure methyl ester B-100 to diesel oil, mixtures of methyl esters are also added to fuel. To be used as fuel, methyl esters must meet certain requirements, one of which is a certain level of acylglycerols. The paper presents results of determination of acylglycerols in diesel oil dotted with fatty acid methyl esters. The compounds were determined by gas chromatography using a high-temperature capillary column DB-5HT, made by J&W, and 1,2,3-tricaproylglycerol as internal standard. The analytical method proposed permits not only determination of acylglycerols, but also differentiation if the FAME added originated from pure vegetation oil or used cooking oil.

## INTRODUCTION

The problems related to the use and production of biofuels have become of increasing importance in view of the consequences of the fact that at present transport practically in 100% of cases depends on petroleum and brings the greatest contribution to the global and local pollution of the environment. The necessity for changes has been reflected in the Directive of the EU Parliament 2003/30/EC of May 8, 2003, in which it is recommended that EU countries should introduce the addition of biofuels in the amount of 2% until 2005, 5.75% until 2010, and 20% until 2020.

Assessment of the suitability of esters produced from vegetable oils (FAME) as fuel is based on the determination of the content of mono-, di-, and triacylglycerols along with free and total glycerol. Their content is one of the most important factors determining the quality of the esters and their possible use as fuel components. The method of glycerine and acylglycerol analysis in the products of transesterification is given in the norm EN-14105:2003 (Fat and oil derivatives). Unfortunately, it applies to pure FAME and can be performed prior to the mixing with diesel oil. Although experimental methods for testing the quality of the mixtures of FAME and diesel oil have been proposed (Wawrzyniak et al. 2005), they do not permit determination of acylglycerol content. The method proposed in this work enables a determination of the contents of acylglycerols in diesel oil dotted with methyl esters labeled as B5, B10, B20, and B50. The determination has been made by gas chromatography with a capillary column enabling separation in high-temperature DB-5HT made by J&W using 1,2,3-tricaproylglycerol (tricaprin) as internal standard. Prior to the analysis, the components had been subjected to derivatization by N-methyl-N-trimethylsilyltrifluoroacetamide (MSTFA).

Another problem is utilization of the used vegetable oils for production of FAME. Presently in economically developed countries including the United States, Canada, and Australia, used vegetable oils are components for production of fodder for animal breeding. Moreover, they are applied in production of soap, fatty acids, and glycerol. In view of the need for biofuel production, it seems that this is the most promising area of utilization of used vegetable oil (Hass et al. 2001; Lee et al. 2002; Agricultural Utilization Research Institute 2002; Sawicki 2005; Zhang et al. 2003). The reuse of used vegetable oil should considerably reduce the cost of FAME production (Sawicki 2005). This possibility has prompted us to find methods of controlling the level of free fatty acids in the product of transesterification and in the diesel oil dotted with FAME.

## ANALYTICAL PART

### The Range of Application

The method is to be applied for determination of mono-, di-, and triacylglycerols in diesel oil admixtured with biofuel components of fatty acid methyl esters. It permits controlling the content of the biocomponent after having been mixed with diesel oil, which is the main advantage of the method over that proposed in EN-14105:2003, allowing determination of the acylglycerols only in pure FAME. The method also permits a differentiation whether the source of a given biocomponent was pure or used vegetation oil.

### Equipment

1. Gas chromatograph equipped with a flame ionization detector (FID) and direct on-column injector (HP 5890 series II)
2. Capillary column with a nonpolar phase of 95% dimethyl-5% diphenyl polysiloxane (we used a DB-5HT column made by J&W of 15 m in length, internal diameter of 0.32 mm, coated with a stationary film phase of 0.1 μm in thickness).
3. Microliter syringes of 10 μL in capacity with fused silica needle (0.18 mm OD, 11.5 cm length)
4. Screw-cap vials with PTFE-faced septa of 10 cm^3^ capacity, microliter syringes of 100 μL, and precision pipette of 1 cm^3^ capacity

### Standards

The standards, n-heptane, and MSTFA used were analytical grade purchased from Supelco and Aldrich.

### Chromatographic Analysis

Chromatographic analysis was performed on a capillary column DB-5HT at the carrier gas (helium) pressure of 50 kPa. The temperature program of the analysis included 50°C held for 1 min, programmed at 15°C/min up to 180°C, programmed at 7°C/min up to 230°C, programmed at 10°C/min up to 390°C, final temperature held for 5 min. The injector and detector temperature was 380°C. The amount of the sample injected on the column was 2 μL.

### Preparation of Standard Solutions and Analyses by Interpolative Internal Standard Method (IISM), Determination of mono-, di-, Triacylglycerols in Diesel Oil

#### Calibration Solutions

The following solutions of the standards in pyridine were prepared: monoolein at 5000 μg/cm^3^; diolein at 5000 μg/cm^3^; triolein at 5000 μg/cm^3^, and the internal standard of tricaprin at 8000 μg/cm^3^. At the next step six calibration solutions containing the above-mentioned solutions in the amounts given in Table 1 were made and placed in vials closed with PTFE membranes.

**TABLE 1:** Composition of the calibration solutions

Calibration solution	0	1	2	3	4	5
μL monoolein solution	0	20	50	100	150	200
μL diolein solution	0	10	20	40	70	100
μL triolein solution	0	10	20	40	70	100

To each calibration solution the portions of 100 μL of the internal standard–tricaprin, 100 μL of diesel oil, and 100 μL of MSTFA were added and the contents were shaken vigorously. After 15 min heptane was added to a volume of 1.2 cm^3^.

#### Preparation of a Diesel Oil Sample

A portion of 100 mg of diesel oil admixtured with FAME was placed in a vial closed with a PTFE membrane. Then, 100 μL of the internal standard and 100 μL of MSTFA were added and the vial was closed and shaken to mix the components. After 15 min 0.9 cm^3^ of heptane was added.

### Identification

**FIGURE 1:**
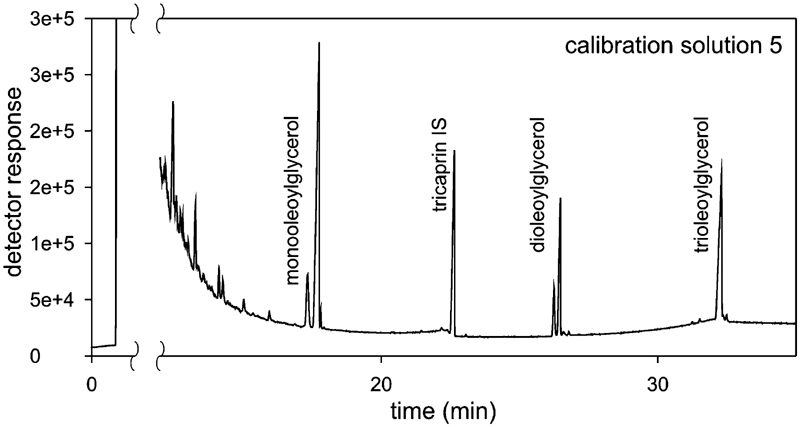
Chromatogram of the calibration solution.

The compounds studied were identified on the basis of the relative retention times, measured with respect to the internal standard (Figure 1). The times were determined on the basis of those obtained from analysis of the calibration solutions. The relative retention times are given in Table 2.

**TABLE 2:** Relative retention times

Compound	Relative retention time
Monoacylglycerols	0.71
Tricaprin	1.00
Diacylglycerols	1.18
Triacylglycerols	1.45

### Calculation of the Regression Parameters of the Calibration Curves

**FIGURE 2:**
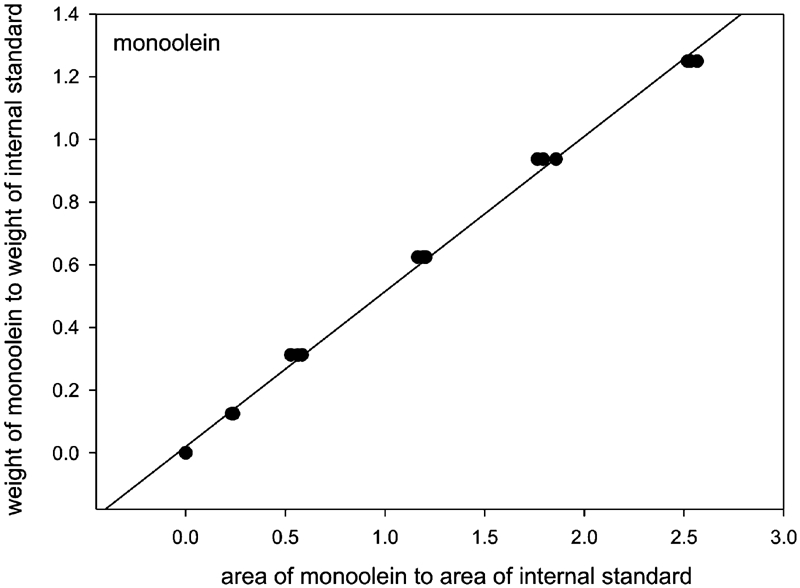
The ratio of the area of the peak corresponding to monoacylglycerols to that of the peak corresponding to the internal standard versus the monoacylglycerols concentration.

Using the calibration solutions, the calibration curves were determined for mono- (Figure 2), di-, and triacylglycerols. For the linear fragments of the curves the regression analysis was done in order to establish the parameters of the regression equation y = ax + b:

where y = M/M_is_; x = A/A_is_

M–the mass of acylglycerol added

M_is_–the mass of the internal standard

A–the area under the peak obtained for a given acylglycerol

A_is_–the area under the peak obtained for the internal standard

The regression line parameters are provided in Table 3.

**TABLE 3:** Linear regression parameters calculated on the basis of the ratio of the area under the peak corresponding to a given acylglycerol to that of the peak corresponding to the internal standard (standard deviation given in parentheses)

Compound	a	b	r (correlation coefficient)
Monoacylglycerols	0.49561 (0.00483)	0.01938 (0.00345)	0.9974 (0.00087)
Diacylglycrols	0.74173 (0.00866)	0.00235 (0.00221)	0.9971 (0.00225)
Triacylglycerols	0.56904 (0.00640)	0.00980 (0.00176)	0.9911 (0.00355)

### Calculation of the Contents of Particular Acylglycerols in the Diesel Oil Admixtured with FAME

Percent contribution of mono-, di-, and triacylglycerols in the diesel oil sample was found from the following formula (1):

(1) $$G_{x}\%=\left[a\left(\frac{A_{x}}{A_{\text{is}}}\right)+b\right]\;\left(\frac{M_{\text{is}}}{\text{m}}\right)\;100$$

where A_x_–area under the peak assigned to a particular acylglycerol (*x*: M–monoacylglycerols, D–diacylglycerols, T–triacylglycerols)

A_is_–area under the peak assigned to the internal standard

M_is_–mass of the internal standard

M–mass of the diesel oil sample.

### Detectability and Determinability

The limits of detectability and determinability of monoacylglycerols in diesel oil were established as 0.001% and 0.003%, respectively; for diacylglycerols as 0.0002% and 0.0007%, respectively; and for triacylglycerols as 0.0007% and 0.002%, respectively.

### Repeatability

The repeatability of the method proposed was assessed on the basis of the calculated standard deviation values of content determinations of particular acylglycerols in the diesel oil containing the biocomponent. The measurements were performed for three series of five independent analyses. Measurements for each series were made on the same day. The results are provided in Table 4. The repeatability estimated as a relative standard deviation varies between 2% and 4%.

**TABLE 4:** Concentrations of particular acylglycerols in a sample of diesel oil admixtured with FAME (B50): Estimation of the method repeatability

Series number	Acylglycerols determined					Mean	Standard deviation	RDS
Monoacylglycerols								
1	0.1608	0.1635	0.1701	0.1760	0.1604	0.1662	0.0067	0.0403
2	0.1596	0.1504	0.1571	0.1596	0.1624	0.1578	0.0046	0.0292
3	0.1612	0.1512	0.1576	0.1594	0.1508	0.1560	0.0048	0.0308
Diacylglycerols								
1	0.0920	0.0935	0.0853	0.0910	0.0868	0.0897	0.0035	0.0390
2	0.0843	0.0878	0.0881	0.0844	0.0799	0.0849	0.0033	0.0389
3	0.0872	0.0912	0.0826	0.0922	0.0897	0.0886	0.0038	0.0429
Triacylglycerols								
1	0.1021	0.1033	0.1000	0.0985	0.0969	0.1002	0.0026	0.0260
2	0.1048	0.1037	0.1022	0.0990	0.1025	0.1024	0.0022	0.0215
3	0.1007	0.0962	0.0965	0.0968	0.1000	0.0980	0.0021	0.0214

### Indirect Precision

The indirect precision was estimated as the relative standard deviation calculated for all results, that is, for the three series of five results. The calculations were made for the same data as those used for the assessment of the method's repeatability (Table 4).

The values obtained are presented in Table 5.

**TABLE 5:** Indirect precision calculated for particular acylglycerols in the diesel oil

Acylglycerols	Mean	Standard deviation	RSD
Mono	0.1600	0.0068	0.0425
Di	0.0877	0.0039	0.0445
Tri	0.1002	0.0028	0.0279

As expected, the indirect precision is higher than the repeatability as the former is affected by a greater number of variables (e.g., the time of measurements).

### Uncertainty

The uncertainty was calculated according to the GUM recommendations (International Organisation for Standarisation 1993). The calculations were performed by the software recommended by GUM (i.e., by GUM Workbrench provided by Metrodata GmbH [http://www.gum.dk]). The concentrations of particular acyglycerols in diesel oil were calculated by the previously described relation Eq. (1).

The calculations were performed for six times repeated measurements for the same B-50 oil sample.

The values of the total and extended uncertainties were estimated taking into regard the effect of the following parameters:

Purity of acylglycerols and the internal standard
Concentration of the standard acylglycerols solutions and that of the internal standard
Volumes of the standard acylglycerol solutions and that of the internal standard
Peak areas assigned to the standards and the internal standard in the calculations of regression parameters
Mass of the diesel oil sample with biocomponents and that of the internal standard taken in determination of acylglycerols in the diesel oil
Peak areas assigned to the acylglycerols and the internal standard in determination of acylglycerols in diesel oil

The values of the extended uncertainty and extension coefficient calculated by the above-mentioned software are given in Table 6.

**TABLE 6:** Values of extended uncertainty (U) and the coefficient of extension (k)

Acylglycerols	Mean	U	K
Mono	0.1570	0.014	2.3
Di	0.0893	0.011	2.3
Tri	0.1050	0.011	2.5

**TABLE 7:** Comparison of the results of measurements with the reference values taking into account the uncertainties

Acylglycerols determined	Reference value x_ref_	Uncertainty for reference material U	|*x* − *x_ref_*|	$2\sqrt{u{\left(x\right)}^{2}+u{\left(x_{\text{ref}}\right)}^{2}}$
Mono	0.165	0.060	0.008	0.089
Di	0.073	0.048	0.016	0.037
Tri	0.149	0.084	0.044	0.089

### Accuracy

The method's accuracy was estimated on the basis of a comparison of the results obtained with the values determined by the gravimetric method in the procedure of preparation of the reference standards of the known content of methyl esters in diesel oil. Because of the lack of the certified reference material, a solution was prepared of a known content of FAME in diesel oil. The FAME used in this solution had an established content of acylglycerols. The uncertainty was also assessed for the reference standard.

The consistence of the results was verified using the following relation:

(2) $$\left|\text{x}-{\text{x}}_{\text{ref}}\right|<2\sqrt{\text{u}{\left(\text{x}\right)}^{2}+\text{u}{\left({\text{x}}_{\text{ref}}\right)}^{2}}$$

When the relation holds, the result is assumed consistent with the reference value.

### Identification of esters originating from cooking oil

The analytical method proposed can also be used for monitoring the level of free fatty acids in diesel oil dotted with FAME. The presence of these compounds testifies to the fact that the esters were obtained from used cooking vegetable oil. The amount of the free fatty acids determined in the diesel oil indicates whether the adequate type of transesterification was applied to decrease the content of these compounds in the final products (esters).

The chromatographic band assigned to free fatty acids is sufficiently intense and characteristic that it can be observed in chromatograms of diesel oil admixtured with FAME to a different degree. Figure 4 presents a chromatogram obtained from determination of acylglycerols in diesel oil dotted with 50% FAME obtained from used vegetable oil. The esters added were obtained by basic transesterification.

**FIGURE 3:**
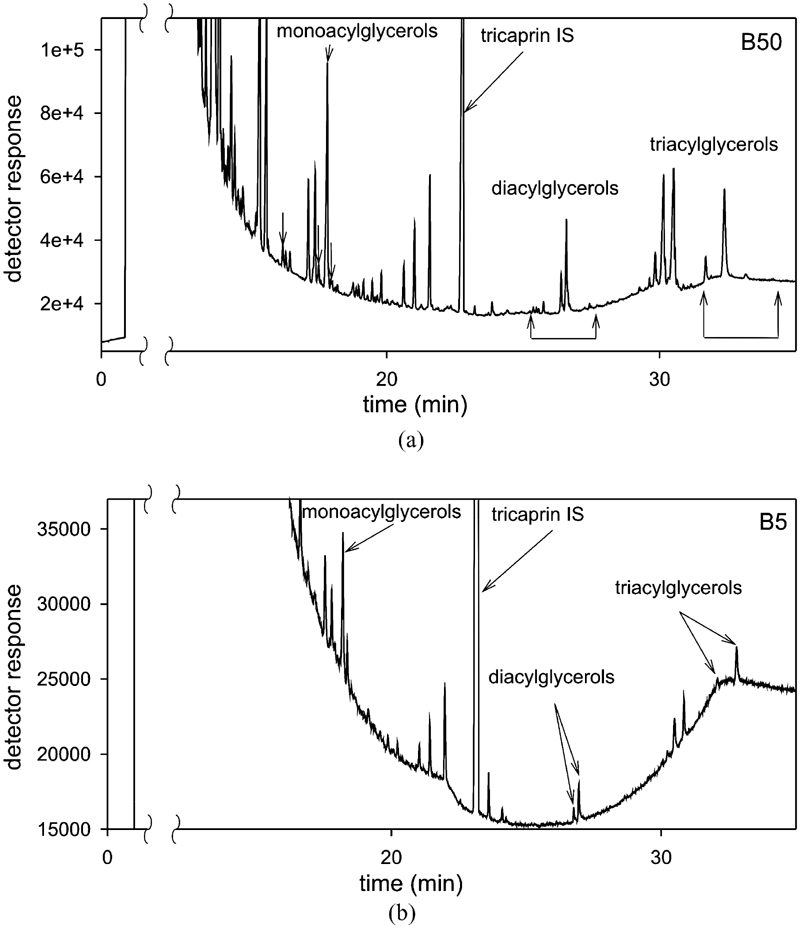
Chromatograms of the diesel oil analyzed B-5 and B-50.

**FIGURE 4:**
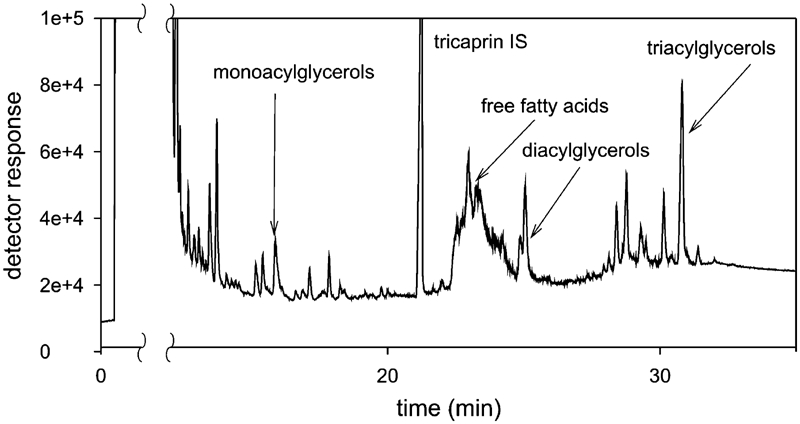
Chromatogram of diesel oil B-50 containing FAME obtained by transesterification of cooking oil.

## CONCLUSIONS

According to the norm EN-14214:2003 (Automotive fuels), the admissible content of monoacylglycerols in the fuel biocomponent is 0.8%, of diacylglycerols is 0.2%, and of triacylglycerols is 0.2%. The limits of determinability of these compounds ensured by the method proposed enable recommendation of the method for controlling the concentrations of acylglycerols in the biocomponent and also in mixtures with diesel oil. The conclusion is valid for the mixtures of FAME/diesel oil at the ratio of 5:95 or higher. Moreover, the method proposed permits detection of free fatty acids in the diesel oil sample admixtured with FAME.
